# Gestational age is related to symptoms of attention-deficit/hyperactivity disorder in late-preterm to full-term children and adolescents with down syndrome

**DOI:** 10.1038/s41598-020-77392-5

**Published:** 2020-11-23

**Authors:** Laura del Hoyo Soriano, Tracie Rosser, Debra Hamilton, Taylor Wood, Leonard Abbeduto, Stephanie Sherman

**Affiliations:** 1 Department of Psychiatry and Behavioral Sciences, MIND Institute University of California Davis, Sacramento, CA USA; 2 Department of Human Genetics, Emory University School of Medicine, Atlanta, GA USA

**Keywords:** Genetics, Psychology, Health care, Risk factors, Signs and symptoms

## Abstract

Attention-deficit/hyperactivity disorder is frequently reported in individuals with Down syndrome, with considerable variation in the expression and severity of the symptoms. Despite growing evidence that gestational age predicts later symptoms of attention-deficit/hyperactivity disorder in the euploid population, this has not been studied in down syndrome. The current study is designed to investigate the influence of gestational age in later symptoms of attention-deficit/hyperactivity disorder in 105 individuals (49 males and 56 females; aged 6–18 years) with Down syndrome who were born at or after 35 weeks gestation. Maternal age at birth, maternal level of education, household income, as well as sex, chronological age, and cognitive level of the participant with Down syndrome were considered in our analysis. Results from this study show that gestational age is related to inattentive and hyperactive/impulsive symptoms in children and adolescents with Down syndrome. Therefore, gestational age should be addressed when considering symptoms of attention-deficit/hyperactivity disorder, as it may have implications for early interventions. More attention is needed toward the advancement of care and follow-up for infants with down syndrome who are born even late preterm or early term.

## Introduction

Attention-deficit/hyperactivity disorder (ADHD) is characterized by inattention, including increased distractibility and difficulty sustaining attention, poor impulse control, and decreased self-inhibitory capacity, motor overactivity, and motor restlessness^1^. A mean worldwide prevalence of ADHD of ~ 2.2% overall (range 0.1–8.1%) has been estimated in the general population aged < 18 years^2^, with a higher prevalence often reported in males^3^. ADHD is also commonly associated with other neurodevelopmental disorders such as down syndrome (DS), with research indicating a mean prevalence of ~ 36.3% overall (range 31–43.9%)^4–6^ and no differences in prevalence between males and females with DS^5^. Most of those with DS and a clinical diagnosis of ADHD are diagnosed as predominantly inattentive^5^. A challenge in interpreting such findings, however, is the extent to which these attention-related symptoms are invariably part of the DS phenotype or are better viewed as secondary comorbid challenges due to ADHD. The high prevalence of comorbid psychiatric conditions in DS^7^ may further complicate the proper diagnosis of ADHD because of the many overlapping symptoms. We propose, therefore, that it may be best to avoid categorical diagnoses and instead investigate dimensions of attention-related problems, characterized as continuous variables^8^, in relation to DS.

Considering the variation in ADHD-like symptoms in the DS population and the high prevalence of other comorbid conditions^9–16^, a key challenge is to identify mechanisms underlying this variability. In this vein, a study of 83 children and young adults with DS found no association between ADHD and age, sex, number of siblings, presence of heart disease, thyroid dysfunction, sleep disorders, or family history of ADHD^5^. Another study showed that ADHD symptoms in youth with DS were related to the dopamine receptor D4 gene on chromosome 11^17^ similarly to the euploid population^18,19^.

Drawing on studies in the euploid population, the association between prematurity and ADHD is well established^20^. Studies have shown that children born early preterm (delivery at gestational weeks < 34) are rated with more symptoms of ADHD, inattention, and hyperactivity/impulsivity than term-born children^21^. Various mechanisms explain the observed association between early preterm birth and ADHD symptoms, all based on a lack of brain development in utero relative to full term babies^22–25^. Some studies have shown that at gestational week 35, the weight of the brain is around 60% of what it will be normally at term^26^. In addition, children born prematurely are at higher risk for postnatal complications and are often exposed to factors that can promote neuronal damage^27^. Even children born late preterm (delivery at gestational weeks 34–36) and early term (delivery at gestational weeks 37–38) score higher on ADHD symptoms later in development compared to children born at gestational week 39 or later^22,23^. Some studies have shown that the negative association between gestational age (GA) and ADHD may be strengthened by factors such as young maternal age, maternal smoking during pregnancy^28^, and low levels of maternal education^23^. Also, sex has been shown to moderate the relationship between GA and ADHD symptoms in euploid preschoolers, with the association appearing to be stronger among girls^21^. Early maternal age at birth (e.g., teenage) has been associated with ADHD symptoms^22^. This same study found an incremental risk for ADHD with each declining week of gestation, even after adjusting for maternal age at childbirth^22^. These findings, which have implications for targeted early interventions in the euploid population, may also have implications for individuals with DS, irrespective of whether they are considered part of the DS-phenotype or a co-occurring condition.

The fetal and neonatal brain in individuals with DS shows deviations from typical development. Whole brain and cerebellar volumes are smaller in DS from 21 weeks gestation, with cortical volumes deviating from that of the typically developing fetus around the third trimester^29,30^. This raises the possibility of even greater susceptibility to the negative effects of preterm and early term birth. The current study was designed to investigate the association between GA and later symptoms of ADHD in DS. The current study investigated this association in 105 children and adolescents with DS while taking into consideration potential confounding factors such as: chronological age (CA), sex, general cognitive level of participants with DS, maternal age at birth, maternal level of education, and family income.

## Methods

### Data source and study sample

Participants and measures reported in the present study are a subset of a larger multicenter project called The Down Syndrome Cognition Project (DSCP)^31–34^, a study of the cognitive and behavioral phenotype of individuals with Down syndrome and the factors influencing phenotypic variability. The study was conducted in accordance with the ethical standards of the relevant national and institutional committees on human experimentation and with the Helsinki Declaration of 1975, as revised in 2008. Each site obtained their Institutional Review Board’s (IRB) approval to conduct the project. The progress of each site was monitored by the Emory Data Coordinating Center.

Participants were recruited through clinics, community events/referral, conferences, advertisements, internet postings, and participation in past research projects. The participating mother provided written consent and participants with DS provided verbal or written assent (when capable) before collecting the data presented in this study.

105 individuals participated in the present study; 49 males and 56 females; aged 6–18 years (M = 11.1 years, SD = 3.4), all for whom English was the primary language spoken at home. Participants were included in the current study if full trisomy 21 had been verified by karyotype, the biological mother was available for participation, and CA of participants with DS was ≤ 18 years. Participants were excluded if they had other chromosomal anomalies, GA prior to 35 weeks, history of epilepsy or other seizure disorder, history of head injury, history of chemotherapy, accidental poisoning, untreated severe hearing or vision loss, or incident of loss of consciousness > 5 min.

### Measures

GA measured in weeks was reported by mothers, as was year of birth of mothers and year of birth of participants with DS. Information about previous ADHD diagnoses, ADHD medication, as well as other comorbid neurodevelopmental disorders, including ASD, was extracted from medical records if available and confirmed by mothers via maternal questionnaire. Maternal age (MA) at childbirth was computed as follows: year of birth of participant with DS–year of birth of mother. We also obtained socio-demographic information via maternal questionnaire as to the self-identified race/ethnicity of the participant with DS (defined by the participating mother), household income, paternal level of education, and maternal level of education.

Symptoms of ADHD were assessed using the Conners Parent Rating Scale, Third Edition (Conners-3)^35^ which reflects criteria for ADHD in the DSM-IV^36^ in individuals aged 6 -18. The Conners-3 contains two primary ADHD subscales (inattentive and hyperactive-impulsive) and other behavioral indices likely to co-exist with ADHD symptoms (i.e., learning problems, executive functioning, defiance/aggression and peer/family relations). In a normative sample, test–retest reliability coefficients ranged from 0.71 to 0.98, with strong discriminant validity between children with ADHD and other groups^37,38^. In a study of 154 children and adolescents with intellectual disability (ID), a Conners Parent Rating Scale total score of 42 provided a sensitivity of 0.9 and a specificity of 0.67 with an area under the curve of 0.84, with strong discriminant validity between children with ID with and without ADHD^39^. The parent version of the Conners-3 also has been used previously in other studies targeting ADHD symptoms of individuals with DS^17,40^, with mean T-scores consistent with diagnostic features of ADHD^40^. For our primary analyses, we used T-scores for the inattentive and the hyperactive-impulsive subscales as the primary symptoms of ADHD, as well as the global index T-score. Higher T-scores for each scale indicate more symptoms of ADHD endorsed by the parent informant.

General cognitive level was measured with the Kaufman Brief Intelligence Test, 2nd Edition^41^. The KBIT-2 assesses verbal and nonverbal cognition across a wide age range (4–90 years old). Our initial outcome was the KBIT-2 Composite IQ score (see Table 2 for descriptive values). However, floor effects emerged; that is, more than 20% of participants’ composite IQ scores were estimated to fall at or below 40 (the lowest possible derived IQ score). For this reason, we computed age-corrected scores from the KBIT-2 composite raw score [(KBIT-2 verbal raw score + KBIT-2 non-verbal raw score)/ 2] as determined by the KBIT-2 scoring manual^41^. The formula used for age-correction was as follows: ((KBIT composite raw score/CA) × 100)^42–44^.This method is similar to that done for the Mullen Scales of Early Learning, another test of general cognition^45^, and, as is true for standard scores, this formula produces different adjusted scores for children of different ages who earn the same raw scores. The KBIT-2 age-corrected score showed no floor or ceiling effects, no correlation with CA (r = 0.1), and a strong correlation with the KBIT-2 IQ score (r = 0.9).

### Data analyses

**Figure 1:**
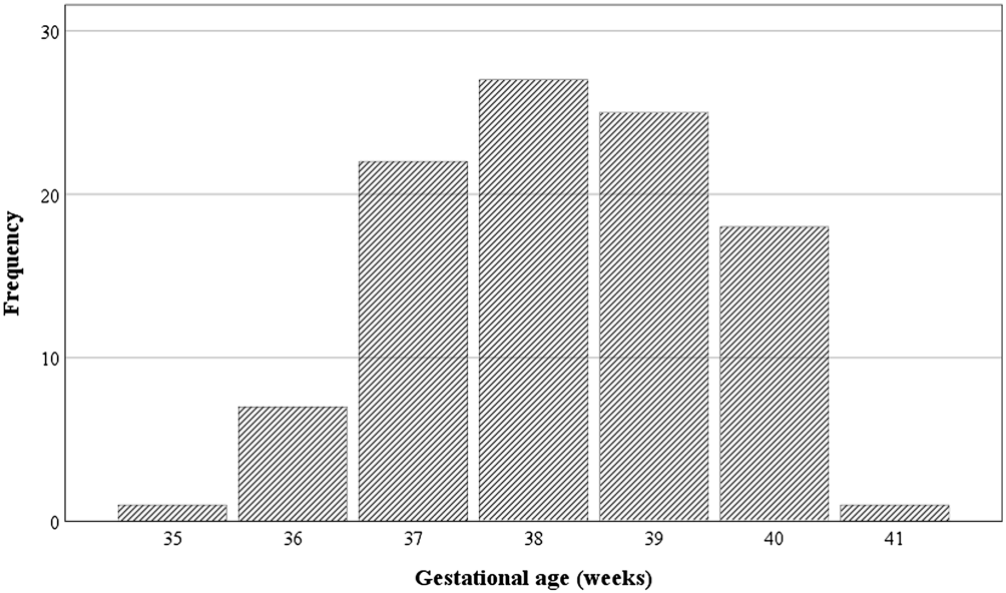
Bar chart representing the distribution of the frequencies of gestational age in the 105 participants with Down syndrome.

The first step consisted of a descriptive analysis of the sociodemographic and clinical parameters. Results are described using central tendency (mean values) and variability (standard deviation and range) for numeric variables, and absolute and relative frequencies for categorical variables in Tables 1 and 2. Figure 1 shows the frequency distribution of GA.

**Table 1:** Demographic Characteristics of Participants with DS.

	Participants (*n* = 105)	
	*n*	%
**Sex**		
Male	49	46.7%
Female	56	53.3%
**ADHD diagnosis**		
Yes	20	19.0%
No	81	77.1%
Missing data	4	3.8%
**ADHD medication**		
No	88	83.8%
Yes	17	16.2%
**Self-identified race/ethnicity**		
African American	8	7.6%
Caucasian	80	76.2%
Other	13	12.4%
Missing data	4	3.8%
**Household income**		
$10,000-$25,000	1	1.0%
$25,000-$50,000	2	1.9%
$50,000–75,000	12	11.4%
$75,000–100,000	18	17.1%
> $100,000	68	64.8%
Missing data	4	3.8%
**Maternal education level**		
Less than high school	1	1.0%
Completed high school or equivalent	2	1.9%
Completed technical school	2	1.9%
Completed 1–3 years of college	11	10.5%
Bachelor’s degree or 4 years of college	47	44.8%
Master’s degree	28	26.7%
Doctoral or professional degree	10	9.5%
Missing data	4	3.8%
**Paternal education level**		
Completed high schoolor equivalent	7	6.7%
Completed technical school	6	5.7%
Completed 1–3 years of college	6	5.7%
Bachelor’s degree or 4 years of college	40	38.1%
Master’s degree	22	21.0%
Doctoral or professional degree	15	14.3%
Missing data	9	8.6%

**Table 2:** Descriptive Statistics of variables of interest.

	*n*	Mean	SD	range
Chronological age at time of ADHD measurement	105	12.4	3.4	6.2–18.9
KBIT-2 Composite IQ score at time of ADHD measurement	105	48.7	10.1	40–86
KBIT-2 Composite (age-corrected) at time of ADHD measurement	105	96.3	53	0–236
Conners-3				
Inattention calculated raw score	105	13.1	6.5	0–29
Inattention T-score	105	65.3	13.1	38–90
Hyperactivity calculated raw score	105	11.4	8.6	0–36
Hyperactivity T-score	105	59.2	12.3	37–90
Global Index calculated raw score	105	8.3	5.4	0–23
Global Index T-score	105	59.1	12.2	36–90
Positive impression Index	105	0.65	0.9	0–4
Negative impression Index	105	0.37	0.7	0–3
Gestational age (weeks)	105	38.2	1.3	35–41
Maternal age at childbirth	101	35.7	4.6	25–47
Paternal age at childbirth	101	37.1	5.6	26–53

In order to decide whether our analyses needed to adjust for potential covariates, we first examined the association between GA and ADHD outcomes (i.e., Inattentive T-score, Hyperactive-Impulsive T-score, and Global Index T-score) for each of the following potential covariates: (1) CA of participant with DS at time of ADHD measurement, (2) KBIT-2 composite age-corrected score at time of ADHD measurement, (3) sex of participant with DS, (4) maternal age at birth, (5) maternal level of education and (6) family income. The computation of all correlations of interest was done using Pearson’s correlation coefficient for numeric variables and ANOVA models for categorical variables. Correction for multiple tests was performed in order to maintain a familywise alpha rate of *p* < 0.05. We then examined the contribution of GA to ADHD outcomes (i.e., Inattentive T-score, Hyperactive-Impulsive T-score, and Global Index T-score) in independent linear regression models. Multiple regression models were adjusted, when necessary, for potential covariates (i.e., if a potential covariate was related (*p* < 0.05) to GA or an ADHD outcome, this covariate was included in the regression model). All predictors were added to each regression model simultaneously. The same analyses were conducted excluding participants taking ADHD medication to see if that impacted our results. Correction for multiple tests was performed to maintain a familywise alpha rate of *p* < 0.05.

Secondary analyses were conducted to rule out a possible link between CA of the participant and parental response bias on the questionnaires. We examined the association between CA of participant and the positive and negative impression scales from the Conners-3. In addition, in order to confirm that higher scores in the Conners-3 were related to ADHD, a one way-ANOVA was conducted with ADHD diagnosis (yes/no) as the independent variable and ADHD outcomes (i.e., Inattentive T-score, Hyperactive-Impulsive T-score, and Global Index T-score) as dependent variables. All the variables included in the models were normally distributed (e.g., skewness (− 1,1) and (− 2,2) for kurtosis). Correction for multiple comparisons was achieved by using the False Discovery Rate (FDR) procedure^46^. All analyses were performed using the statistical software package SPSS (Version 18.0; SPSS Inc., Chicago, IL, USA).

### Ethical approval

The authors assert that all procedures contributing to this work comply with the ethical standards of the relevant national and institutional committees on human experimentation and with the Helsinki Declaration of 1975, as revised in 2008. Informed consent was obtained from the parent or guardian of each participant before testing.

## Results

Table 1 provides the demographical information about our study sample. Twenty of the 105 participants (19%) had a previous diagnosis of ADHD. Of those 20, 17 (16.2%) were taking ADHD medication. See supplementary Table 1 for co-occurrence of other neurodevelopmental disorders.

We found no association of ADHD outcomes or of GA with maternal age at birth, maternal level of education, family income or sex of participants with DS. However, CA of participants with DS at time of ADHD measurement was related to T-scores for the Inattentive subscale (R = − 0.25, *p* = 0.01) and the Hyperactive-Impulsive subscale (R = − 0.25, *p* = 0.01), as well as the Global Index of the Conners-3 (*p* = 0.001, R = − 0.32). CA was not related to the positive (R = − 0.08, *p* = 0.4) or the negative impression scores (R = 0.008, *p* = 0.9). In addition, the KBIT-2 composite age-corrected score at time of ADHD measurement was related to the Global Index of the Conners-3 (R = − 0.21, *p* = 0.04). See supplementary Table 2 for details. After controlling for multiple comparisons, only the relationship between CA and the Global Index of the Conners-3 remained significant. However, for a conservative approach, CA and general cognitive functioning at time of ADHD measurement were both included as covariates in the corresponding regression models reported below.

**Figure 2:**
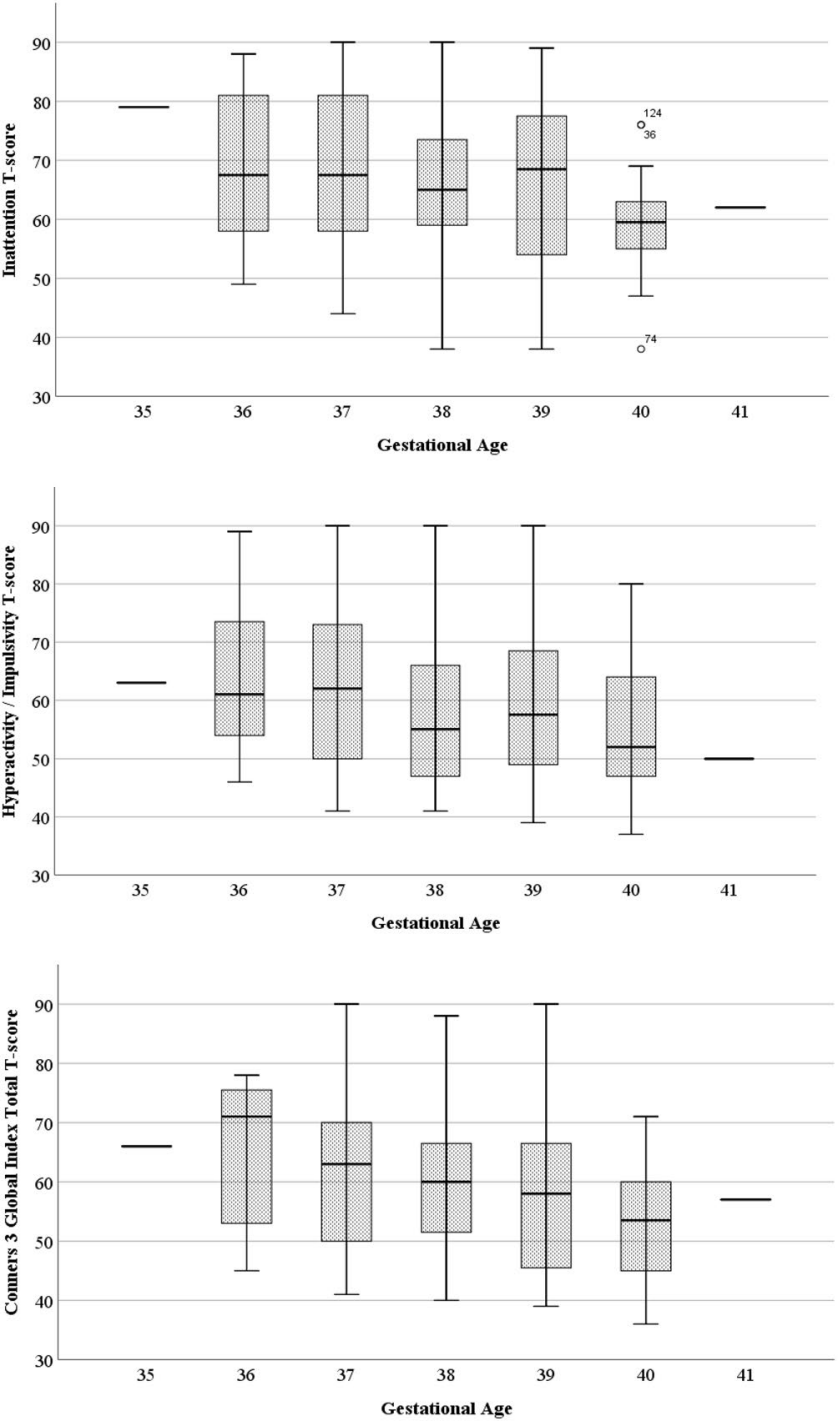
Simple box plot representing the distribution of ADHD outcomes in relation to gestational age in the 105 participants with Down syndrome.

As seen in Table 3, multiple regression models show that GA is associated with T-scores for the Inattentive and Hyperactive-Impulsive subscales both before and after adjusting for CA of participants with DS at time of ADHD measurement. In addition, GA is associated with the Global Index of the Conners-3, before and after adjusting for CA, and the KBIT-2 composite age-corrected score at time of ADHD measurement. As represented in Fig. 2, lower GA is related to higher T-scores for ADHD measures from the Conners-3. The contribution of GA to T-scores from the Conners-3 excluding those participants taking ADHD medication showed similar results to those reported in the entire sample (see Table 4).

**Table 3:** Gestational age associated with ADHD symptoms while adjusting for demographic factors: results from stepwise regression analyses in the 105 participants with DS.

Explanatory variable	β	Adjusted *R*^2^ of full model	*p* value (crit.)	Dependent variable
**Step 1**				Inattention T-score
Gestational age	− .205	.033	.036 (.05)	
**Step 2**				
Gestational age	− .199	.073	.037 (.05)	
Chronological age	− .220		.022 (.03)	
**Step 1**				Hyperactivity/impulsivity T-score
Gestational age	− .215	.037	.030 (.04)	
**Step 2**				
Gestational age	− .210	.085	.029 (.03)	
Chronological age	− .239		.014 (.02)	
**Step 1**				Global Index T-score
Gestational age	− .217	.037	.030 (.03)	
**Step 2**				
Gestational age	− .220	.160	.019 (.02)	
Chronological age	− .286		.003 (.005)	
KBIT-2 composite score (age corrected)	− .202		.033 (.04)	

Chronological age and KBIT-2 composite score of participants with Down syndrome are at time of ADHD measurement. FDR-adjusted critical values are presented next to *p* values, if a *p* value is below or at the critical value it remains significant after controlling for multiple comparisons.

**Table 4:** Gestational age associated with ADHD symptoms while adjusting for demographic factors: results from Stepwise Regression Analyses excluding those participants with DS taking ADHD medication (n = 88).

Explanatory variable	β	Adjusted *R*^2^ of full model	*p* value (crit.)	Dependent variable
**Step 1**				Inattention T-score
Gestational age	− .205	.033	.036 (.05)	
**Step 2**				
Gestational age	− .199	.075	.037 (.05)	
Chronological age	− .226		.018 (.02)	
**Step 1**				Hyperactivity/impulsivity T-score
Gestational age	− .215	.046	.030 (.04)	
**Step 2**				
Gestational age	− .210	.110	.029 (.03)	
Chronological age	− .252		.009 (.01)	
**Step 1**				Global Index T-score
Gestational age	− .217	.037	.030 (.03)	
**Step 2**				
Gestational age	− .220	.160	.019 (.02)	
Chronological age	− .286		.003 (.005)	
KBIT-2 composite score (age corrected)	− .202		.033 (.04)	

Chronological age and KBIT-2 composite score of participants with Down syndrome are at time of ADHD measurement. FDR-adjusted critical values are presented next to *p* values, if a *p* value is below or at the critical value it remains significant after controlling for multiple comparisons.

Secondary analyses focused on the correlation between having a diagnosis of ADHD and the Conners-3 total and subscale scores are reported in Supplementary Table 3. Participants with a reported diagnosis of ADHD had higher T-scores for the Inattentive subscale, the Hyperactive-Impulsive subscale and the Global Index of the Conners-3 compared to those who did not have an ADHD diagnosis.

## Discussion

The present study is unique in assessing association of GA at birth with ADHD-like symptoms at 6 to 18 years of age in a group of 105 individuals with DS. This retrospective study found that an earlier GA (limited to those with GA of 35 weeks or more) was associated with more symptoms of ADHD among children and adolescents with DS, after adjusting for CA and general cognitive functioning at time of ADHD measurement. Our results are in line with those studies of the euploid population showing that children born early term as well as those born late preterm scored higher on ADHD symptoms compared to children born at gestational week 39 or later^22,23^. In the general population, various mechanisms have been proposed to explain the association between preterm birth and future neurodevelopmental difficulties. First, the immaturity of the brain relative to full term-expectations observed in the early weeks of gestation (i.e., ≤ 35 weeks) has been proposed as the main mechanism involved in long-term difficulties, including ADHD^21,26^. However, typically developing infants born even at a late preterm GA are at increased risk for morbidities in the immediate newborn period, including a higher rate of respiratory distress syndrome and transient tachypnea of the newborn when compared with term infants^47,48^. These problems could, in turn, lead to volumetric losses in specific brain regions and may partially explain the long-term difficulties related to ADHD symptoms^21^.

In the fetus with DS, differences in brain development and altered regional brain growth have been detected from 21 weeks of gestation when compared to controls; reduced cerebellar volume apparent in the second trimester, and significant alteration in cortical growth becoming evident during the third trimester, have also been reported^29^. Thus, we speculate that the effect of early GA on an already altered brain seen in DS may be greater than that for the euploid population. This would partly explain why we still see an effect of GA in long-term ADHD symptoms in our sample of late-preterm to full-term participants. Nevertheless, further longitudinal studies are needed to confirm this association and to examine the consequences of trisomy 21 molecular and behavioral levels. In contrast to the euploid population, the association between GA and long-term ADHD symptoms in DS was not more pronounced with inattention than with hyperactivity^21,49,50^. One reason for this difference might be the influence of trisomy 21 on brain development and its vulnerability to early GA. It could also be that we are not observing this differential pattern due to a lack of participants born early preterm and very early preterm in our sample relative to previous studies in the euploid population included^21,49,50^. Future studies should explore whether GA effects on ADHD symptoms in children and adolescents with DS by including those born before the 35th week of gestation.

Interestingly, the CA of the participants with DS was also related to ADHD outcomes. Our results showed that younger participants generally scored higher on inattentive, hyperactive, and general ADHD symptoms when compared to older participants. This finding is in line with previous research in DS^51,52^ and in the general population^53^. Taken together, these results suggest that although ADHD is chronic in nature, symptoms may present in different ways or in varying degrees as an individual moves through life stages^54^. One possible explanation may be that those with ID whose behavioral difficulties have been addressed over the years will have a range of learned strategies to use when ADHD-like symptoms become problematic^55^. However, it is important to note that our results are based on cross-sectional data and only participants from 6 to 18 years of age are included in our sample. In addition, we have no information on whether these families and their children have interventions to manage behavioral and cognitive difficulties typically observed in DS. Therefore, further longitudinal studies are needed to confirm the hypothesis that ADHD symptoms decrease over time in individuals with DS.

Importantly, the relationship between GA and later ADHD symptoms was still significant after adjusting for CA at time of ADHD measurement, suggesting that GA contributes to the later ADHD symptoms in addition to CA at time of ADHD measurement. Because the effect size of GA limited to 35 weeks and older was small, it remains to be determined whether the contribution of GA to later ADHD symptoms is clinically meaningful. However, these preliminary findings strongly suggest that more studies are needed that include participants with GA below 35 weeks to better to understand the full impact of early GA.

As expected, GA was not related to CA at time measurement, which rules out a potential confounding effect with ADHD symptoms. Therefore, this study adds to the growing body of evidence that indicates more attention needs to be paid toward the care and follow-up of infants born preterm, even those between GA of 35–39 weeks, perhaps even more so for those with DS.

The fact that the degree of cognitive delay was not related to the main symptoms of ADHD suggests that hyperactivity, impulsivity and inattention-related symptoms in those with DS are not a consequence of the ID; therefore, ADHD difficulties may be best conceptualized as comorbid challenges. However, it is important to note that although general cognitive functioning was not related to subscales of hyperactivity, impulsivity or inattention, it was related to the CA of the participants with DS. Indeed, CA explained more of the variance in the outcomes of the Conners-3 than did GA. These results suggest that symptoms of inattention and hyperactivity/impulsivity as reported by parents are more severe in younger individuals with DS. Our results are in line with the statistics reported from the Centers for Disease Control and Prevention (CDC) stating a higher percentage of ADHD diagnoses for children aged 6–11 compared to those aged 12–17^56^. Note, however, that the CA of participants with DS in our study was not related to the negative or the positive impression score from the Conners-3. Therefore, the link between CA and the Conners-3 rating is not explained by a response bias in the parents. However, further longitudinal investigations are needed to understand the link between CA and ADHD symptoms in DS including different methods and environments of assessment.

Other factors included in the present study that have been shown to be likely to contribute to ADHD outcomes in the euploid population did not show a correlation in our study sample. These include sex and maternal age at childbirth. One reason may be related to the characteristics of our sample. For example, maternal age at birth of the participating mothers was between 25 and 45 years of age, and GA was ≥ 35 weeks. Previous studies have reported a link between younger maternal age at birth and later ADHD symptoms^28,57^; however, we had few mothers in the younger age range and thus would not have the power to identify this association. In addition, those studies showing a link between maternal age at birth and GA have included babies who were born before the 35th week of gestation^58^. Another interesting result of our study was the fact that the presentation and severity of ADHD symptoms was not related to sex, which is in line with previous research in DS^51,52^, but differs from the typically developing population^59^. In addition, the lack of association between the level of general cognitive functioning and the severity of ADHD symptoms is also in line with previous research conducted in individuals with DS^4^. Therefore, although attention deficits and the symptomatology associated with ADHD appear with greater frequency in people with DS^4,11,60–62^, ADHD-related symptoms in this population are not better explained by the degree of ID. Taken together, our results suggest the need for similar monitoring of ADHD symptoms in both males and females with DS, regardless of the level of ID, to ensure appropriate health care.

### Limitations

This study has several limitations. First, different methods are used to determine GA, for example, prenatal ultrasound or date of last menstrual period^63^. Bias may have occurred if the source of information to estimate GA is associated with both exposure (GA) and outcome (ADHD symptoms). Second, participants in the present study are part of a larger study (DSCP) in which GA < 35 was an exclusion criterion; including participants with GA < 35 would have increased our ability to examine the broader impact of fetal immaturity for those with DS. Our current results most likely underestimate this impact. Third, we relied on maternal reports of ADHD symptoms, which are not equivalent to a psychiatric evaluation. Finally, the current study is based on a cross-sectional evaluation of ADHD symptoms in relation to CA at time of ADHD symptom measurement.

## Conclusion

In summary, findings from the current study are promising but need to be considered as preliminary, suggesting that GA plays a role in the later emergence of ADHD symptoms in children and adolescents with DS. Therefore, more attention must be focused on the advancement of care and follow-up for infants with DS who are born even late preterm or early term. Further longitudinal studies including other perinatal (e.g., fetal lung maturity, biophysical profile, birth weight, Apgar score), other methods of ADHD assessment and behavioral measures (e.g., Behavior Assessment Scale for Children), as well as including individuals with GA < 35, are needed to further explore the effects of fetal immaturity in future development for those with DS.

## Supplementary information

Supplementary Tables.

## Data Availability

The datasets used and/or analyzed during the current study are available from the corresponding author on a reasonable request.
