# Supplementary material for: Gestational age is related to symptoms of attention-deficit/hyperactivity disorder in late-preterm to full-term children and adolescents with down syndrome
Source: Sci Rep. 2020 Nov 23;10:20345. doi: 10.1038/s41598-020-77392-5 (PMC7683733; doi:10.1038/s41598-020-77392-5)
Supplement: Supplementary file 1 — Supplementary Tables. [file 41598_2020_77392_MOESM1_ESM.docx]

Gestational age is related to symptoms of Attention-Deficit/Hyperactivity Disorder in late-preterm to full-term children and adolescents with Down syndrome

Laura del Hoyo Soriano*^1^, Tracie Rosser^2^, Debra Hamilton^2^, Taylor Wood^1^, Leonard Abbeduto^1^, and Stephanie Sherman^2^

^1^ MIND Institute University of California Davis, Department of Psychiatry and Behavioral Sciences, Sacramento, CA, USA

^2^ Emory University School of Medicine, Department of Human Genetics, Atlanta, GA, USA

*** Correspondence:** [ldelhoyo@ucdavis.edu](mailto:ldelhoyo@ucdavis.edu)

**Supplementary Table 1.** Descriptive values regarding the presence of other neurodevelopmental disorders.

|  | Participants with DS (*n*=105) | |
| --- | --- | --- |
|  | *n* | % |
| Autism spectrum disorder |  |  |
| Yes | 8 | 7.6% |
| No | 93 | 88.6% |
| Missing data | 4 | 3.8% |
| Disruptive behavior |  |  |
| Yes | 8 | 7.6% |
| No | 94 | 89.5% |
| Missing data | 3 | 2.9% |
| Anxiety disorder |  |  |
| Yes | 4 | 3.8% |
| No | 45 | 42.9% |
| Missing data | 55 | 52.4% |
| Depression |  |  |
| Yes | 0 | 0% |
| No | 91 | 86.7% |
| Missing data | 14 | 13.3% |
| Obsessive compulsive disorder |  |  |
| Yes | 3 | 2.9% |
| No | 49 | 46.7% |
| Missing data | 52 | 49.5% |
| Seizure disorder, Epilepsy or Infantile spasms (treated) | | |
| Yes | 0 | 0% |
| No | 105 | 105% |
| Missing data | 0 | 0% |

**Supplementary Table 2:** Association between GA and ADHD symptoms rated by parents with the Conners-3 (Inattentive T-score, Hyperactive-Impulsive T-score, and Global Index T-score) with each of the potential covariates.

|  | | Inattention T-score | Hyperactivity/  Impulsivity T-score | Global Index  Total T-score | Gestational age |
| --- | --- | --- | --- | --- | --- |
| Family income^2^ | F | .513 | 1.47 | .666 | .964 |
|  | p-value | .674 | .226 | .575 | .413 |
| Maternal educational level^2^ | F | 2.07 | .831 | .657 | .898 |
|  | p-value | .117 | .484 | .583 | .449 |
| Maternal age at birth^1^ | r² | -.100 | -.061 | -.142 | -.009 |
|  | Sig. | .334 | .559 | .167 | .930 |
| KBIT-2 Composite (age corrected)^1^ | r² | -.149 | -.154 | -.209^*^ | -.018 |
|  | p-value | .149 | .140 | .042 | .859 |
| Chronological age of participant with DS^1^ | r² | -.250^*^ | -.255^*^ | -.324^**^ | -.008 |
|  | p-value | .012 | .011 | **.001** | .940 |
| Sex of participant with DS² | F | 2.57 | .768 | 1.78 | .086 |
|  | p-value | .111 | .383 | .185 | .770 |

The computation of all correlations of interest was done using Pearson’s correlation coefficient (r²) for numeric covariables^1^ and One-Way ANOVA model for categorical covariables². Bolded p-values are those which remained significant after correcting for multiple comparisons.

**Supplementary Table 3**: Association between previous diagnosis of ADHD with ADHD symptoms rated by parents with the Conners-3 (Inattentive T-score, Hyperactive-Impulsive T-score, and Global Index T-score). Results from One-Way ANOVA model comparing Conners-3 outcomes between those with and without an ADHD diagnosis.

|  | Descriptive means | | One-Way ANOVA | | |
| --- | --- | --- | --- | --- | --- |
|  | ADHD | NO ADHD | Mean S. | F | p values |
| Conners 3 Global Index Total T-score | 68.4 | 56.9 | 2102.6 | 16.2 | .000 |
| Hyperactivity/Impulsivity T-score | 67 | 57.4 | 1395.2 | 8.9 | .004 |
| Inattention T-score | 73.2 | 63.2 | 1487.9 | 9.6 | .003 |
